# The Influence of Measurement Conditions on the Electrocaloric Effect in Ferroelectric Ceramics of Modified Barium Titanate

**DOI:** 10.3390/ma17133329

**Published:** 2024-07-05

**Authors:** Magdalena Krupska-Klimczak, Krzysztof Ziewiec, Irena Jankowska-Sumara

**Affiliations:** 1 Institute of Security Studies and Computer Science, University of the National Education Commission, Podchorążych 2, 30-084 Kraków, Poland; magdalena.krupska-klimczak@up.krakow.pl; 2 Department of Exact and Natural Sciences, University of the National Education Commission, Podchorążych 2, 30-084 Kraków, Poland; 3 Institute of Technology, University of the National Education Commission, Podchorążych 2, 30-084 Kraków, Poland; krzysztof.ziewiec@up.krakow.pl

**Keywords:** electrocaloric effect, electrocaloric strength, barium titanate

## Abstract

In this work, the electrocaloric effect (ECE) and electrocaloric strength (Δ*T*/*E*) were measured and thermal and dielectric studies were performed on Pb-modified BaTiO_3_ (BPT). The saturated hysteresis loops and normal ferroelectric behavior of the ferroelectric ceramics allow the utilization of the indirect method to estimate the electrocaloric properties. The electrocaloric measurements were performed under high (18 kV/cm) versus low (8 kV/cm) electric field conditions. These conditions were chosen to notice and then eliminate an artificial negative electrocaloric effect in the tested ceramics. At the same time, relatively high values of positive electrocaloric temperature change Δ*T* (~ 2.19 K) and electrocaloric strength Δ*T*/*E* (~0.27–0.11 K·cm/kV) were obtained.

## 1. Introduction

The perovskite-type compound of pure barium titanate as well as the solid solutions based on it are well-known ferroelectric materials that are widely used in many applications. For the practical use of BaTiO_3_ (as a capacitor, piezoelectric material, PTC resistor, etc.), a high relative permittivity is required over the widest possible operating temperature range. Usually, some additives are needed to tailor the physical and electrical properties of BaTiO_3_ to meet the requirements of various applications (e.g., [1]). Much attention has been paid to the solid solutions of BaTiO_3_ (BT) with PbTiO_3_ (PT). Both compounds are ferroelectrics with tetragonal symmetry at room temperature, which, however, have significantly different Curie temperatures: 403 K (BaTiO_3_) and 763 K (PbTiO_3_). By mixing these two ferroelectrics, the Curie temperature can be shifted almost in a linear manner [2]. The application advantages of the compound thus formed have been described in many publications [3,4,5,6]. Ba_1−x_Pb_x_TiO_3_ (BPT) solid solution with 0 < x < 0.1 is mainly used as a Positive-Temperature-Coefficient Thermistor (PTCR) working at high temperatures [7,8]. To sum up, (Ba_1−x_Pb_x_)TiO_3_ (BPT) ceramics are technologically important due to their applications in electrics and mechatronics as ceramic capacitors, piezoelectric transducers, and actuators.

It appears that the compounds based on BaTiO_3_ may also be of interest for solid-state refrigeration techniques because of their significant electrocaloric effect (ECE) [9,10,11,12,13]. In a paper by Moya [9], it was reported that the temperature variations for pure single crystal BaTiO_3_, measured in both direct and indirect ways, reached 1 K. Recent experimental [14,15,16] and theoretical [17,18] studies also revealed the presence of a negative ECE, which is sometimes also called inverse or abnormal and describes the decrease in the temperature when an electric field is applied. In those publications, it was explained that if the external electric field is not parallel to the spontaneous polarization of the material, the dipolar entropy can increase under adiabatic conditions, causing a negative ECE. The effect of negative ECE can also result from the situation when the applied electric field is not enough to fully saturate the electrical polarization [19]. An artificial (apparent) negative ECE appears then. The possibility of enhancing and controlling the ECE in BaTiO_3_ by different dopants was also studied. Several authors mainly considered heterovalent doping as the possibility to enhance and control the ECE in BT materials through the presence of internal defect dipoles [14,15,16,20,21]. Fewer works are devoted to isovalent substitutions such as those that produce a more or less stoichiometric solid solution avoiding the additional polarization coming from defect dipoles. In this work, we focused on determining the ECE in a solid solution of the mentioned BPT. Since obtaining good quality ceramics of this type of material possesses some difficulties due to the high vapor pressure of lead at high temperatures during the synthesis process, we chose commercial ceramic PIC110 from Lederhouse Germany [22]. This piezoelectric compound is generally described as a modified barium titanate material with a Curie temperature of 423 K.

To investigate the effect of the electric field on the appearance and disappearance of a negative ECE, the measurements were carried out under conditions of the so-called high and low electric field for two samples of different thicknesses.

## 2. Materials and Methods

The main purpose of the study was to determine the electrocaloric temperature change Δ*T* (using the indirect method described in detail in [23,24]) and electrocaloric strength Δ*T*/*E* in the modified barium titanate ceramic, from the PI Ceramic company [22], marked as PIC110. To achieve this goal, ferroelectric hysteresis loops and specific heat as a function of temperature were measured. For a better description of the selected basic properties of this ferroelectric ceramic, it was decided to add microstructural and dielectric measurements.

The imaging of surface morphology and chemical analysis was performed using a JEOL JSM-6610LV Scanning Electron Microscope (Tokyo, Japan) equipped with an Energy-Dispersive X-ray (EDS) detector manufactured by Oxford Instruments (Abingdon, UK). Measurements were carried out in secondary electron image mode at an accelerating voltage of 20 kV and a working distance of 10 mm.

Differential Scanning Calorimetry was used for measurements of the specific heat of the PIC110 sample. The sample, with a weight of 35 mg, was placed in an aluminum crucible. The Netzsch DSC F3 Maia (Selb, Germany) scanning calorimeter operating in an argon atmosphere at a 40 mL/min flow rate for the temperature range from 120 to 500 K was used. For the cooling and heating processes, a constant rate of 10 K/min was applied. 

To meet the desired high and low electric field conditions, two samples (A and B) of different thicknesses were prepared by cutting them from a ceramic block. Thus, electrical measurements were performed on two ceramic samples with the same surface ~3.5 mm^2^ and a thickness of 1 mm (sample A) and 0.485 mm (sample B). The upper and lower surfaces of the ceramic samples were coated with silver electrodes and placed in the silver furnace. Computer-controlled measurements, during which the temperature was controlled by a thermocouple with an accuracy of 0.1 K, were performed on heating with a rate of 1 K/min, from room temperature, up to a few kelvins above phase transitions. The dielectric characterization of PIC110 was performed with the use of a Precision LCR meter (Keysight, Santa Rosa, CA, USA). The measurements were performed on heating and cooling in the frequency range from 200 Hz to 2 MHz. The ferroelectric hysteresis loops (for the purpose of electrocaloric temperature change calculations) were obtained using the standard Sawyer–Tower method in the quasistatic limit [25]. The amplitude of the electric field varied from 0 to 8×10^5^ V/m for sample A and between 0 and 18×10^5^ V/m for sample B, and the frequency of the test signal was set at 30 Hz. The loops were collected on heating and cooling processes with an increment of 1 K.

## 3. Results and Discussion

### 3.1. Microstructural Characterization

**Figure 1:**
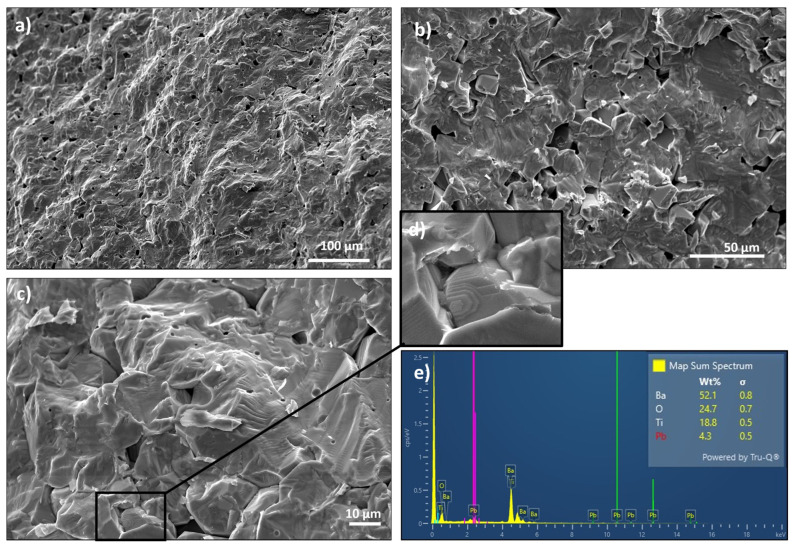
Scanning Electron Microscopy (SEM) images of the microstructure of fractures of PIC110 (**a**–**c**), a step-structure terrace (**d**), and map sum spectrum (**e**). The images (**a**–**c**) were taken at a magnification of (**a**) 200×, (**b**) 500×, and (**c**) 1000×.

Figure 1 presents SEM micrographs of PIC110 taken at a magnification of (a) 200×, (b) 500×, and (c) 1000×. A step-terrace structure is visualized in Figure 1d. 

One can see that the surfaces of fracture run on both the grain and the intergranular boundaries. The sample is dense with well-developed grains, which indicates a good sintering of the ceramics. The estimated size of a single grain is around 9 μm whereas the porosity was estimated at the level of ~5%. We also present an EDS analysis from the surface which is visualized as a spectrum in Figure 1e. It indicates that PIC110 is a BaTiO_3_ modified mostly by means of Pb doping (other dopants in small quantities, however, cannot be excluded). Based on the EDS spectrum, the atomic percentage of the Pb in BaTiO_3_ was established at about ~3.5%.

### 3.2. Identification of the Phase Transitions

**Figure 2:**
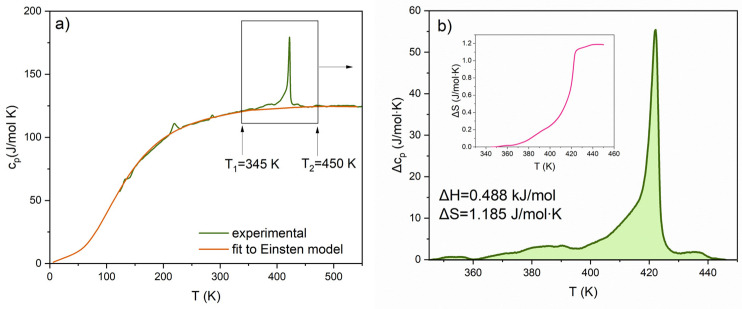
Temperature dependencies of (**a**) the specific heat and (**b**) excess specific heat in the temperature range marked by the rectangle in figure (**a**) for PIC110. The insert in (**b**) presents the temperature dependence of entropy change (Δ*S*).

The measurement of specific heat in a wide temperature range of 120–500 K is presented in Figure 2a. The solid red line (the so-called baseline) represents the fitting of experimental data to the Einstein model given by Equation (1):

(1) $$c_{v}=3N_{A}K_{B}{\left(\frac{\Theta_{E}}{T}\right)}^{2}\frac{e^{z}}{{\left(e^{z}-1\right)}^{2}}$$

where:

$$z=\frac{\Theta_{E}}{T}$$

$$c_{v}=c_{p}-R$$

The anomalies associated with the phase transitions that exist in pure BaTiO_3_ (according to [26]) are at temperatures as follows: 393 K for Cubic–Tetragonal (C-T), 278 K for Tetragonal–Orthorombic (T-O), and 183 K for Orthorombic–Rhombohedral (O-R). It can be noticed that the highest anomaly manifested at 422 K in Figure 2a is related to the main phase transition (C-T) and is shifted towards a higher temperature when compared to pure BaTiO_3_. The lower transition (T-O), on the other hand, is moving toward lower temperatures. The lowest phase transition (O-R) is invisible on our *c*_p_(*T*) run. 

In Figure 2b, the excess specific heat (Δ*c*_p_(*T*)) is shown, which is defined as a difference between experimental data and the fitted lattice heat capacity (baseline). Through the integration of Δ*c*_p_(*T*) between the temperature limits *T*_1_ and *T*_2_ (as marked in Figure 2a) and using Equations (2) and (3) [27], the enthalpy (Δ*H*) and the entropy change (Δ*S*) were calculated and are as follows: ~0.488 kJ/mol and ~1.185 J/mol∙K, respectively. Such a small value of entropy change is usually related to the phase transitions of the displacive type which is typical of the ferroelectric BaTiO_3_.

(2) $$∆H=\int ∆c_{p}\left(T\right)dT,\;\;\;\;\;$$

(3) $$∆S=\int \frac{{∆c}_{p}}{T}dT.$$

**Figure 3:**
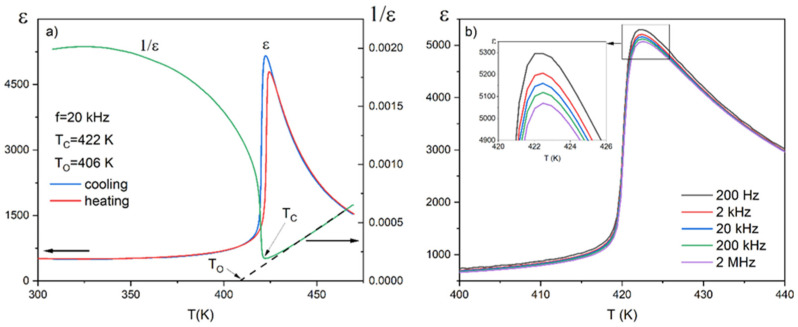
Temperature dependences of (**a**) the dielectric constant ε of one cycle (heating and cooling) with the reciprocal of the electric permittivity 1/*ε* (f = 20 kHz) and (**b**) the dielectric constant *ε* for a set of frequencies in the range 200 Hz–2 MHz for PIC110.

The results of dielectric studies for PIC110 are shown in Figure 3. Figure 3a presents the temperature dependence of electric permittivity *ε* (*T*) for one chosen frequency (20 kHz) on heating and cooling. The *ε* (*T*) characteristics show a typical ferroelectric behavior with an anomaly at *T*_C_ (at 422 K for the cooling process, which is in perfect agreement with the value obtained from calorimetric measurements and that provided by the manufacturer) and indicate the I order of the phase transition associated with the existence of temperature hysteresis. The temperature run of the reciprocal electric permittivity 1/*ε*(*T*) presented in the same figure (green line) also points to the first-order phase transition. However, a small discrepancy from the Curie–Weiss law (see Equation (4)) just above *T*_C_ can be observed. Based on the temperature dependence of 1/*ε* it was possible to indicate the Curie–Weiss temperature *T*_O_ = 406 K. By using Equation (4), the estimated Curie constant *C* is of the order of 8×10^5^ K, which again indicates that the phase transition in this compound is mainly of the displacive type, similar to that in pure barium titanate.

(4) $$\varepsilon =\frac{C}{T-T_{O}}$$

In Figure 3b, a small dielectric dispersion in the measured frequency range (200 Hz–2 MHz) can be observed, but without the features of relaxation behavior. The delicately rounded maximum of the electrical permittivity at *T*c indicates a slight diffusing of the phase transformation compared to pure BaTiO_3_. It should be noted that the values of *ε* presented in this paper agree with those presented in [2,28], where the authors describe the linear relation between the Curie temperature and the molar ratio of BaTiO_3_ vs. PbTiO_3_. It is also worth noting that the addition of Pb into BaTiO_3_ slightly lowers the *ε*_max_ value compared to the pure BaTiO_3_ ceramic.

### 3.3. Ferroelectric and ECE Measurements

The main purpose of this work was to investigate the ECE in this compound, which has not yet been measured for this material. Thus, there appears to be an opportunity to complete and better characterize this commercial ceramic. The indirect method in the Materials and Methods section requires the measurement of the ferroelectric hysteresis loops within a specific temperature range. The details of this method were described in our previous papers [29,30].

**Figure 4:**
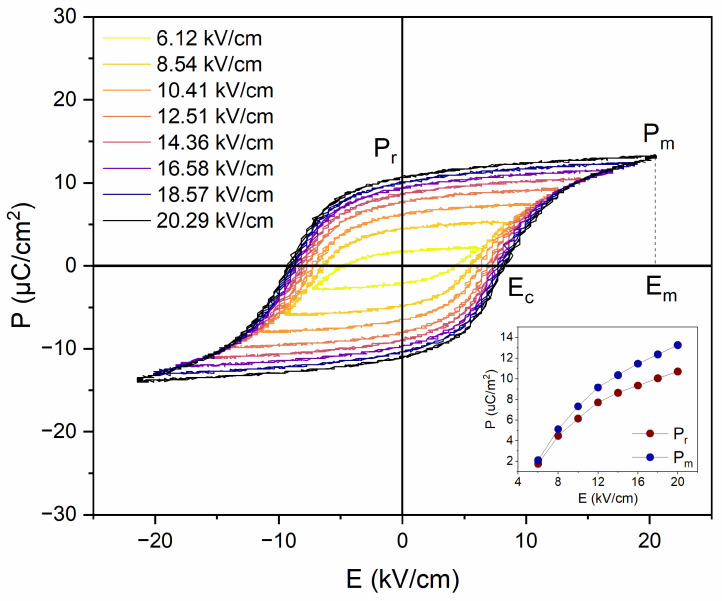
The ferroelectric hysteresis loops measured at RT for sample B under the application of different values of the electric field between 0 and 20 kV/cm. The inset shows *P*_r_(*E*) and *P*_m_(*E*) dependence.

Figure 4 presents a set of ferroelectric hysteresis (P-E) loops of PIC110 measured at 30 Hz for the applied electric field in the 6–20 kV/cm range. From the figure shown, it appears that at room temperature the hysteresis loops start to be saturated above an electric field of 8 kV/cm. The characteristic ferroelectric parameters (*E*_c_—coercive field, *P*_r_—remnant polarization, *E*_m_—maximum electric field, and *P*_m_—maximum polarization) for each electric field applied were retrieved and are shown in Table 1. In the inset of Figure 4, the *P*_r_(*E*) and *P*_m_(*E*) dependences are also presented.

**Figure 5:**
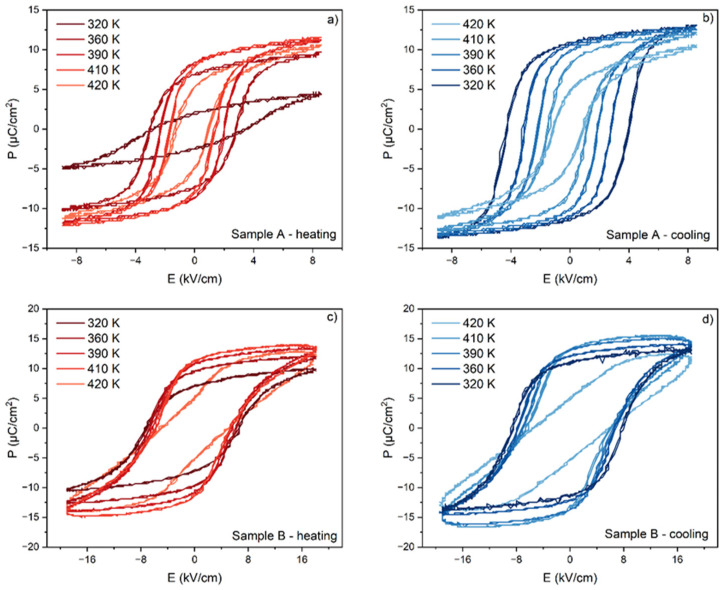
The ferroelectric hysteresis loops at selected temperatures measured for samples A (**a**,**b**) and B (**c**,**d**) during the heating (red lines) and cooling process (blue lines).

In the next step, ferroelectric hysteresis loops were measured for both samples A and B as a function of temperature from room temperature to several degrees above the phase transition. A comparison of the temperature evolutions of these loops for the heating and cooling process is shown in Figure 5 for both samples. It is worth noting here that the thicknesses of both samples were selected so that the maximum voltage applied to the sample would give a field of 8 kV/cm for sample A and 18 kV/cm for sample B. There is a difference between loops recorded on heating and cooling. In particular, in the case of sample B, subjected to a much higher electric field, the effect of electrical conductivity on the shape of the loops can be seen.

**Figure 6:**
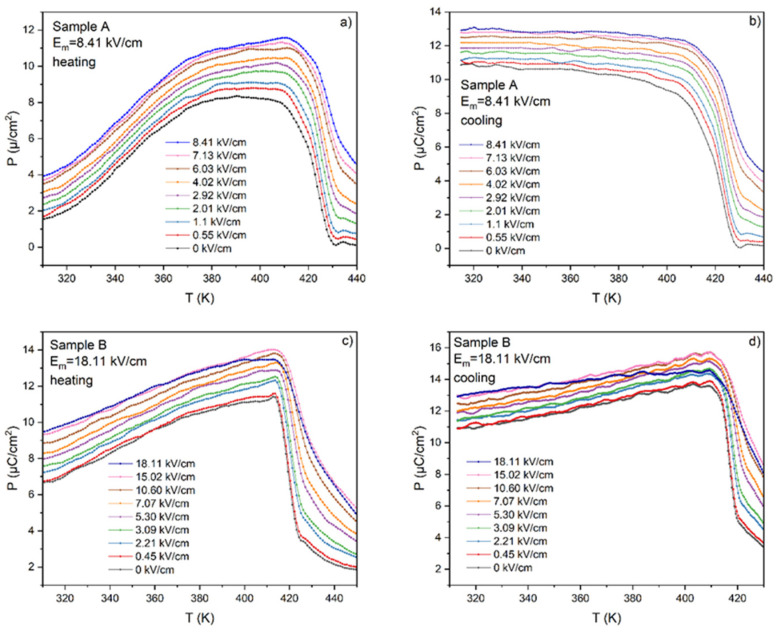
The temperature dependence of polarization for samples A (parts **a** and **b**) and B (parts **c** and **d**) during the heating (**a**,**c**) and cooling process (**b**,**d**).

The whole sets of the ferroelectric hysteresis loops were used to develop the temperature and field dependence of polarization for both samples (A and B) for heating and cooling processes, as shown in Figure 6. Focusing on Figure 6a (sample A heating), we can see a difference in the *P*(*T*) dependence between heating and cooling runs. Namely, a very slight increase in polarization in the initial range of heating (from RT up to ~350 K) can be observed. Then, the polarization increases quite abruptly until temperature *T* = 416 K (from *P*_m_ = 3.9 µC/cm^2^ to *P*_m_ = 11.6 µC/m^2^ for *E* = 8 kV/cm), and finally above *T* = 420 K it quickly decreases. The observed rapid decrease in polarization is connected with the ferroelectric–paraelectric phase transition. During cooling, a similar relationship can be observed around the temperature of the phase transition; however, contrary to heating, we can notice an almost constant polarization value from up to room temperature.

In the case of sample B, the shape of *P*(*T*) dependences are similar during the heating and cooling processes although higher polarization values can be observed on cooling.

**Figure 7:**
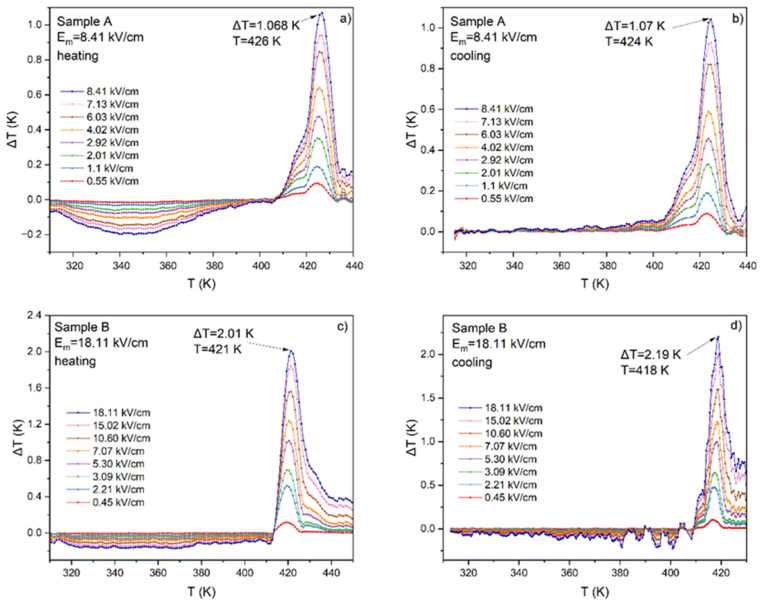
Temperature dependence of electrocaloric temperature change (Δ*T*) measured for sample A (**a**,**b**) and sample B (**c**,**d**) during heating (**a**,**c**) and cooling (**b**,**d**).

Using the Maxwell relationship between polarization, specific heat, and electric field, the electrocaloric temperature change Δ*T* was calculated using Equation (5) and is presented in Figure 7 as a function of temperature for both samples A and B. The procedure of calculations was the same as that described in Ref. [23].

(5) $$∆T=-\frac{1}{C_{p}\rho }\int_{E_{1}}^{E_{2}}T{\left(\frac{\partial P}{\partial T}\right)}_{E}dE$$

The results of the calculation of the ECE for samples A and B are presented in Figure 7. One can notice that higher values, i.e., Δ*T* = 2 K, were achieved for sample B—a sample under the influence of a higher electric field. The value of Δ*T* = 2 K can already be considered a so-called giant effect. The fact that higher values of Δ*T* are obtained under the application of a higher electric field is well known and needs no explanation. Another interesting fact is the existence of a small negative ECE for sample A. Such behavior can be explained by incomplete polarization in small electric fields. Although both samples were under the electric field that exceeds the coercive field, the mobility of the dipoles is too small at low temperatures to be switched along the electric field direction. At higher temperatures, the mobility of dipoles increases and dipole switching occurs much more easily. At the same time, polarity switching occurs more easily when using a larger electric field and the polarization reaches higher values. On the other hand, the influence of electrical conductivity on the value and shape of the hysteresis loops for the thick sample A and thin sample B is clearly visible.

**Figure 8:**
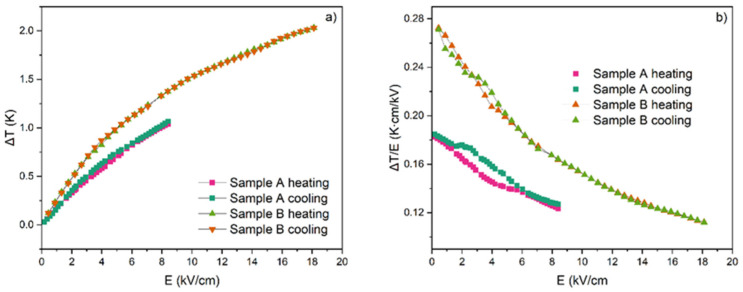
Electrocaloric temperature change (**a**) and electrocaloric strength (Δ*T*/*E*) (**b**) as a function of applied electric field measured for sample A (*E*_m_ = 8 kV/cm) and sample B (E_m_ = 18 kV/cm) and for the heating and cooling processes at the temperature showing the maximum value of Δ*T*.

Even though 2 K is an excellent and even gigantic result for ferroelectric ceramics, it should be remembered that this is still an absolute value. Quoting absolute ECE values is not reliable when it comes to the characteristics of the material. More illustrative in this regard is to present the value of Δ*T* as a function of the applied electric field and the electrocaloric strength Δ*T*/*E*. The Δ*T*(*E*) and Δ*T*/*E*(*E*) dependences for the Low *E* and High *E* as well as for the heating and cooling processes are given in Figure 8.

Comparing the Δ*T*(*E*) dependences for samples A and B (Figure 8a), one can observe that sample A reaches lower values of Δ*T* than sample B for the same value of *E*. Moreover, the course of curves tends to achieve saturation and for sample A, this saturation was estimated at the level of ~1.07 K whereas for sample B it was as high as ~2.3 K. In Figure 8b, the electrocaloric strength Δ*T*/*E* as a function of the electric field *E* is presented for both samples. Δ*T*/*E* for sample A changes from 0.17 to 0.12 K·cm/kV and for sample B from 0.27 to 0.11 K·cm/kV.

The typical Δ*T*/*E* values for BaTiO_3_, both pure and doped (by various ions such as La^2+^, Ca^2+^, Dy^2+^, Sn^4+^, Zr^4+^), are in the range of 0.01–0.075 K·cm/kV (0.1–0.75 K·mm/kV) [31]. It cannot be denied that our results are almost 10 times higher. Thus, our results indicate promising properties of Pb-modified BaTiO_3_ ceramics from the point of view of the application of the electrocaloric effect. More systematic measurements for PBT solid solution ceramics in a wider range of compositions would thus be desirable.

### 3.4. Influence of Measuring Conditions on the ECE Effect

**Figure 9:**
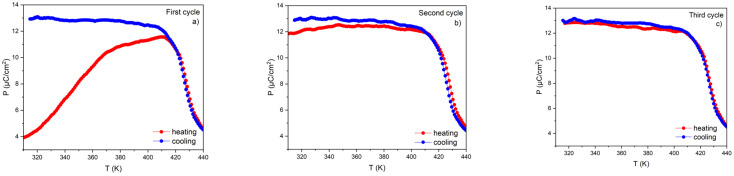
The temperature dependence of polarization for sample A measured during three consecutive heating (red) and cooling (blue) cycles. (**a**) first cycle (**b**) second cycle (**c**) third cycle.

At this study stage, we checked the effect of successive heating and cooling cycles of ceramics in the presence or absence of an electric field and their influence on the polarization values and the resulting ECE. For this purpose, we chose sample A (the thicker one), which was exposed to the electric field for three successive heating and cooling cycles. An AC electric field with an amplitude of 8 kV/cm and a frequency of 30 Hz was maintained throughout the experiment. The results of the experiment are presented in Figure 9. 

**Figure 10:**
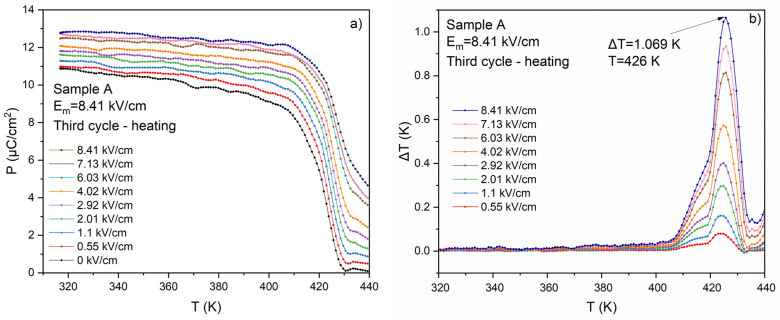
The temperature dependence of polarization (**a**) and electrocaloric temperature change (**b**) for sample A after three cycles of heating and cooling.

This experiment shows that the sample undergoes gradual polarization as early as the third cycle. The final effect is similar to that observed in sample B, i.e., that subjected to a high electric field. The *P*(*T*) dependence and calculated ECE for sample A after the third cycle are presented in Figure 10. It is easy to notice that the negative electrocaloric effect initially observed for this sample disappears after the third cycle, while the positive effect remains at the same level.

Annealing the sample at a temperature above T_C_ and cooling it without an electric field result in a return to the pre-experimental situation, i.e., the situation of the first cycle.

The experiment described in this paragraph unambiguously indicates that the measured negative ECE in the tested sample is certainly related to the incomplete polarization of the sample, which was polarized in an electric field that was too low, even though the electric field exceeded the value of the coercive field.

## 4. Conclusions

The thermal, ferroelectric, and electrocaloric properties of PIC110, a commercially available modified BaTiO_3_ ceramic, were investigated. The results of specific heat and dielectric studies indicate a slightly diffusing I-order phase transition at *T*_C_ = 422 K (as declared by the producer). The goal of this paper, on the one hand, was to measure the ECE in a ceramic sample of modified BaTiO_3_, and, on the other hand, to test the poling effects on the occurrence or non-occurrence of a negative ECE.

PIC 110 reveals excelled electrocaloric temperature change Δ*T* around 2 K for the relatively low electric field (18 kV/cm) and excellent electrocaloric strength Δ*T*/*E* with a value of the order of 0.11 to 0.27 K·cm/kV. This places the tested ceramic among promising materials for electrocaloric applications.

In this paper, we also tested the effect of the appearance of the artificial negative ECE. There are many reports in the literature [32] about the true negative effect that can be observed in antiferroelectrics, some relaxors, ferroelectrics near FE-FE phase transition, some axial ferroelectrics, etc. However, some cases of negative effects are due to the insufficient polarization of the ceramics when measuring the ECE using the indirect method. For this reason, we investigated the problem of negative effect occurrence in the investigated samples and identified the measuring conditions for which the negative effect disappears. 

There is no doubt that the indirect method appears to be easy to use, and is a convenient method for the investigation of the electrocaloric effect in ferroelectric ceramics. It does not differ from the direct method in terms of accuracy, but it should be used with particular caution and the correctness of the negative effect, if it occurs, should always be checked.

Considering the possible application of the ECE in cooling devices, it should be kept in mind that electrocaloric strength (Δ*T*/*E*), i.e., the ratio of the electrocaloric temperature change Δ*T* and applied electric field *E*, is independent of the dimensions or the geometrical shape of the investigated material. In, e.g., ref. [31], there can be found information about the values of Δ*T*/*E* obtained for different kinds of samples (bulk, thin films, and others). It is generally known that despite great interest in thin films (due to the extremely high values of Δ*T* obtained in experiments [31]), much higher values of Δ*T*/*E* are obtained for large-scale materials. For this reason, hopes for possible electrical cooling are still tied to bulk materials. Thus, a commercially available piezoelectric ceramic, PIC 110, which is based on ferroelectric BaTiO_3_, can be used as the electrocaloric element which makes it potentially useful for practical application as an microrefrigerator element.

In light of the above, Pb-modified BaTiO_3_ materials are worthy of further investigations, i.e., more detailed ECE research—including direct ECE—in order to develop near-room-temperature electrocaloric cooling devices—would be desirable. The modification of their composition by examining other values of the Pb admixture to obtain the highest possible Δ*T* with a simultaneously high Δ*T*/*E* will be highly desirable.

## Figures and Tables

**Table 1:** Values of maximum polarization *P*_m_, remnant polarization *P*_r_, and coercive field *E*_c_ for different electric fields obtained from ferroelectric hysteresis loops measured for PIC110 at room temperature.

*E*_m_ (kV/cm)	*E*_c_ (kV/cm)	*P*_r_ (μC/cm^2^)	*P*_m_ (μC/cm^2^)
6.12	4.23	1.77	2.10
8.54	5.57	4.47	5.12
10.41	6.38	6.15	7.33
12.51	6.89	7.71	9.16
14.36	7.32	8.64	10.36
16.58	7.71	9.35	11.47
18.57	7.93	10.06	12.37
20.29	8.24	10.71	13.26

## Data Availability

The original contributions presented in the study are included in the article, further inquiries can be directed to the corresponding author.
